# Physical Activity Promotion in the Preschool Years: A Critical Period to Intervene

**DOI:** 10.3390/ijerph9041326

**Published:** 2012-04-16

**Authors:** Gary S. Goldfield, Alysha Harvey, Kimberly Grattan, Kristi B. Adamo

**Affiliations:** 1 Healthy Active Living & Obesity Research Group, Children’s Hospital of Eastern Ontario Research Institute, Ottawa, ON K1H 8L1, Canada; Email: ggoldfield@cheo.on.ca (G.S.G.); aharvey@cheo.on.ca (A.H.); kgrattan@cheo.on.ca (K.G.); 2 Department of Pediatrics, University of Ottawa, Ottawa, ON K1N 6N5, Canada; 3 School of Human Kinetics, University of Ottawa, Ottawa, ON K1N 6N5, Canada; 4 School of Psychology, University of Ottawa, Ottawa, ON K1N 6N5, Canada; 5 Department of Psychology, Carleton University, Ottawa, ON K1S 5B6, Canada

**Keywords:** preschoolers, physical activity, obesity, prevention, critical period

## Abstract

The primary aim of this paper is to provide a rationale for the necessity of intervening with a physical activity intervention in the preschool years and why the daycare environment is amenable to such intervention. We also review the prevalence of physical activity, sedentary behaviour and obesity in the preschool population and the impact that these lifestyle behaviours and conditions have on the health of preschool aged children, as secondary objectives. Moreover we discuss implications for intervention and research using a “lessons learned” model based on our research team’s experience of conducting a randomized controlled trial aimed at increasing physical activity, reducing sedentary behaviour and improving motor skill development and body composition in preschoolers. Lastly, we make conclusions based on the literature and highlight issues and directions that need to be addressed in future research in order to maximize health promotion and chronic disease prevention in the pediatric population.

## 1. Introduction

Canadian children are very inactive, with only 9% of boys and 4% of girls meeting the new Canadian PA Guidelines of accruing 60 minutes of moderate-to-vigorous physical activity (MVPA) every day. Moreover, Canadian children are getting an average of 6 hours or more of screen time each day [1]. This high level of sedentary behaviour is concerning, given screen time is associated with elevations in risk factors for type 2 diabetes in children and youth, and these associations are independent of body composition and physical activity [2,3,4]. Similarly, considerable interest exists around the rise in child obesity rates in Westernized countries and its adverse impact on psychosocial health and chronic disease, which is becoming increasingly prevalent in childhood [5,6]. The good news is that systematic reviews have shown that increasing physical activity (PA) reduces the risk of these chronic diseases by improving cardio-metabolic profile in children [7] and may also help prevent child obesity, especially in young children [8]. Participation in PA may also improve motor skills which can influence participation in PA activities and sport later in life. Given that PA and sedentary behaviours track from early childhood to adulthood [9], and obesity is difficult to treat once it develops [10], increasing children’s PA levels during the early years may alter their activity trajectory and increase the likelihood they will be physically active throughout development and into adulthood, which would reduce the risk of chronic diseases related to inactivity.

The primary aim of this paper is to provide a rationale for the importance of intervening with a physical activity intervention in the preschool years and to establish the daycare environment as an appropriate and amenable target for such intervention. We also review the prevalence of physical activity, sedentary behaviour and obesity in the preschool population and the impact that these lifestyle behaviours and conditions have on the health of preschool aged children, as secondary objectives. Moreover we discuss implications for intervention and research using a “lessons learned” model based on our research team’s experience of conducting a randomized controlled trial aimed at increasing PA, reducing sedentary behaviour and improving motor skill development and body composition in preschoolers. Lastly, we offer directions for future research in the early years as a critical period in order to maximize health promotion and chronic disease prevention in the pediatric population.

## 2. Physical Activity and Health in Preschoolers

According to the long-standing physical activity guidelines from the U.S.-based National Association for Sport and Physical Education [11], preschool aged children should accumulate 120 minutes of physical activity per day, with 60 minutes coming from structured physical activity and 60 minutes from unstructured or spontaneous active play. In addition, NASPE guidelines state that preschoolers should not be sedentary for more than 60 minutes at a time (except when sleeping). More recent physical activity guidelines from the UK [12] and Canada call for 180 minutes of physical activity per day [13].

Many parents perceive their children to be active [14], and in particular, there is a common societal belief that preschool children (3–5 yrs) are by nature, highly active. In contrast, the bulk of evidence from studies that have directly measured PA in preschoolers has illustrated that very few children meet PA recommendations (see review [15]) set forth by NASPE, with only a few small studies showing otherwise [16,17]. Regardless of how it is measured or reported, minutes/hour [18,19,20], minutes/day [21,22,23], percentage of time [24,25,26,27,28,29,30], levels of MVPA in preschoolers have, in the majority of cases, been shown to be consistently low and levels of sedentary behaviour are high. Studies have reported that 3–5 year olds spend a small proportion of their time (anywhere between 10 and 100 minutes/day depending on sampling frame, and cut-offs) engaged in MVPA accounting for only a small fraction of the day [15,31,32,33]. A recent study of Canadian preschoolers in *family-based* care also found remarkably similarly low levels of MVPA [20], that include 12.7 minutes of MVPA per day and 276.5 minutes of sedentary behaviour per day while in the child care environment. The bulk of available evidence suggests that young Canadian children are insufficiently active to accrue the health benefits of physical activity and therefore may be at risk for adverse health consequences related to physical inactivity.

From the cardio-metabolic standpoint, the currently available evidence, albeit sparse, indicates that PA during the preschool years is associated with: (i) more desirable body composition [34,35,36,37,38,39,40,41], and (ii) decreased cardiovascular risk factor status (*i.e.*, lipid profile, insulin resistance) [42,43,44] and lower sub-maximal heart rate during exercise [45]. Furthermore, motor development, or the process by which a child acquires movement patterns and skills, has also been shown to be positively associated with PA [23,46,47,48,49]. This is important as motor skills are a key factor in the likelihood of participation in various forms of PA during later childhood and adolescence [46,50,51].

## 3. Prevalence of Obesity in Preschool Aged Children and Its Relation to Health

The prevalence of child obesity has increased dramatically over the past three decades in North America and has now reached epidemic proportions. It is important to note that recent evidence from epidemiological research dispels the widely held societal belief that preschool-aged children are healthy and weight related issues do not begin this early in development. For example, Canadian surveillance data, using directly measured heights and weights gathered as part of the Canadian Community Health Survey, indicate that overweight and obesity exist in the preschool age group with 15.2% of children aged 2 to 5 years categorized as overweight and 6.3% as obese [52]. Overweight children have higher risks for numerous health conditions and children who become obese before the age of 6 are likely to be obese later in childhood [53]. The negative trajectory continues as these children often remain overweight as adults [54]. In addition to the adverse medical complications of obesity that can manifest in childhood (insulin resistance, type 2 diabetes, non-alcoholic fatty liver disease *etc.*), there is a heavy psychosocial and economic burden that obesity related diseases have on the health care system. 

## 4. Day Care as a Critical Setting/Environment for Intervention

The landscape of childcare in the developed world has changed dramatically over the last 2 decades, with the vast majority of children now attending some form of daycare during their preschool years. More than half of Canadian children between the ages of 6 months and 5 years are enrolled in some form of non-parental care, averaging 29 hours/week in this arrangement [55,56]. In keeping with the settings-based approach to health promotion, which acknowledges the influence of place on behaviour [57], it is believed that powerful influences on children’s PA levels are the social and physical environments in which they spend time [25,30,58]. As such, the paid child daycare setting provides an ideal opportunity to emphasize the adoption of a physically active lifestyle by enhancing the PA behaviours and movement skills of preschool-aged children which may mitigate the decline in activity often seen during the transition from childhood to adolescence [59]. There is a body of evidence that suggests that in group preschool and child care settings, policies and practices strongly influence children’s PA [18,25,58]. Although intervention to modify PA in the child care setting is still in its infancy, a recent review of the relevant preschool intervention literature identified the potential for structured PA programs to increase the amount and intensity of PA and improve motor skills [60] and concluded with a call for increased research in child care settings to identify what strategies are most appropriate to produce active, healthy children. Only one small study has reported on the quantity of PA accrued by Canadian preschoolers outside of parental care [20] which illustrated that preschoolers in a family-based child care setting (*i.e.*, in a home where typically one adult cares for ≤7 children at one time) engaged in very minimal amounts of PA and considerable sedentary time over the course of a day. Knowing that activity levels decline as children age, with data showing this decline starting at age 4 yrs, it is important to target the preschool age group in attempts to mitigate this reduction [61].

A 2010 review has drawn attention to the issue of consistently low levels of objectively measured PA in the preschool years [29] and specifically addressed the need to evaluate interventions to promote PA in child care settings. In addition, a pertinent review paper by Timmons and colleagues [62], has called for further research focusing on knowledge gaps in this critical period. The Australian Department of Health and Ageing commissioned a consortium paper [63] that specifically addressed the need ‘*for the development, implementation and evaluation of simple, well designed interventions to promote PA and reduce screen time in children during the early years*’. Additionally a 2009 systematic review looking at the efficacy of intervention to improve motor development in young children highlighted the limited quantity and quality of interventions and recommended that ‘*both educators and researchers should be involved in the implementation of an intervention and that parental involvement is critical to ensuring transfer of knowledge from the intervention setting to the home environment*’ [64]. As for high quality randomized controlled trials (RCTs) testing lifestyle interventions in preschool-aged children, in addition to the two ongoing that we are aware of [65,66], there are not many. The published RCTs [67,68], including those summarized in a recent systematic review by Timmons and colleagues [69], have yielded mixed results. Inconsistency in results may be related to methodological differences and variations in the intervention protocol. However, the 2012 review by Kreichauf and colleagues [70] found evidence that increasing PA by 30 minutes a day in the day care setting was effective in improving body composition, thereby aiding obesity prevention in later childhood.

The appeal and advantage of intervening with child care providers to promote PA within the daycare setting is the potential wide scale public health benefits that can be accrued from increased PA. Given more than 50% of Canadian children alone between the ages of 6 months and 5 years are enrolled in some form of day care, averaging about 6 hours per day in this arrangement [55,56], many young children can be positively impacted by health promotion efforts associated with intervening in day care settings, similar to the rationale for school-based intervention. 

## 5. Rationale for Intervening in the Preschool Years: A Critical Period to Intervene

PA is an acknowledged component of a healthy lifestyle and childhood experiences as it has an important impact on lifelong behaviour and health. There are many evidenced-based reasons for targeting intervention designed to increase PA and reduce sedentary behaviour in the preschool years, making it a potential critical period to intervene. For instance, as previously described, the majority of 3–5 year-old children spend an average of 6 hours each day in daycare setting, PA and aerobic fitness track throughout development [71], aerobic fitness has been shown to be a strong predictor of longevity and is inversely related to all cause mortality [72] and PA in the preschool years has associated with numerous health benefits outlined earlier. Thus intervening with daycare providers in this setting, to equip them with the knowledge and abilities to promote healthy active living in children under their care, may provide substantial public health benefits.

While PA has been shown to be helpful in the management of child obesity [73], it appears that PA plays a more important role in preventing rather than treating child obesity [74]. Although not extensive, several studies show converging evidence that PA is associated with more desirable body composition [34,35,36,37,38,39]. Relatedly, a comprehensive narrative review, critically evaluating educational strategies promoting PA in the preschool setting in the context of obesity prevention, concluded that studies reporting positive outcomes implemented PA sessions that lasted at least 30 minutes per day [70]. Moreover, they reported that the existence or installation of playground marking or fixed play equipment had no effect, whereas the presence of the addition of portable play equipment was positively correlated with moderate-to-vigorous physical activity. To overcome time constraints, the authors suggested to integrate PA into the daily routines and other areas of the preschool curriculum.

It should be noted that in addition to the medical burden of obesity, there is a heavy concomitant economic burden on our health care systems. For example, treating obesity related medical conditions has been estimated to cost about $4.3 billion in Canada [75]. £5.1 billion in the UK [76] and US$75 billion in the United States [77]. Data from the Longitudinal Study of Australian Children indicate that for all children aged 4 and 5 in 2004–2005, those who were overweight had a combined 5-year Medicare bill that was AUD$9.8 million higher than that of normal weight children. These statistics reveal that the financial burden to the public health system of childhood overweight and obesity occurs even during the preschool years [78]. While there are many critical periods for intervention over one’s lifespan, intervening early in life by promoting PA may not only help prevention of chronic disease but mathematical modeling suggests that targeted interventions for young children (0–6 yrs) could yield considerable cost savings [79].

Opportunities for PA and motor development in early childhood may, over the lifespan, influence the maintenance of a healthy active lifestyle for health promotion and disease prevention. It has been noted that children with low movement competence usually exhibit low PA levels [80,81] and tend to be vigorously active less often, play less on large playground equipment and spend less time interacting socially with their peers [80]. Fundamental movement skills (e.g., catching, throwing, jumping, running *etc.*) are the essential building blocks for the acquisition of more refined and complicated skills that can be applied later in life, such as sporting, recreational and physical activity pursuits [82,83,84]. However, movement skills will not develop to their full potential without opportunities to practice in environments that are stimulating and supportive [85,86]. Butcher and Eaton [81] found that preschoolers movement competence was already influencing the PA choices and levels of preschoolers. Thus, targeting improved fundamental and gross motor skills in the preschool years may lead to greater participation in PA, sports, and organized activities and the associated health benefits of these active pursuits. Consistent with this hypothesis, successful development of motor skills has been shown to provide a stimulus for ongoing PA engagement that contributes to short- and long-term health benefits [87].

One potential advantage of targeting improvements in healthy active living behaviours in the early years is that altering health behaviours in young children is considerably easier than in older children, adolescents, or adults [10]. This is likely due to the fact that young children’s health behaviour is more malleable as they have either not yet adopted an unhealthy lifestyle or haven not had one for a long period of time. Perhaps more importantly, it is easier to promote PA in young children because they are completely responsive to environmental (daycare provider/parent) control of the eating and activity environment. Moreover, care providers, including parents are the most powerful role models and therefore can represent intermediaries and targets for intervention to foster healthy habits in their young children. To the best of our knowledge, we are not aware of any research examining the relationship between physical activity patterns in parents and preschool-aged children. However, findings from studies in older children are encouraging. In a sample of children aged 8–12 years, Kalakanis and colleagues [88] found a strong relationship between duration and patterns of PA in parents and children. Similarly, Fuemmeler and colleagues [89] found that greater parental MVPA as measured by accelerometry was associated with increased child MVPA, and having two parents with higher levels of MVPA was associated with greater levels of activity in children. Relatedly, Golan *et al.* (2004) found that treating the parents as the exclusive agents of change was more effective in fostering lifestyle changes resulting in weight loss compared to treating preadolescent overweight children alone [90], and these effects were maintained at 7-year follow-up [91]. Moreover, Golan found that targeting only parents as the agent of change resulted in more favourable changes in eating and activity behaviours than the standard family-based approach to child obesity treatment that included a parent and child [92]. Taken together, there is converging evidence that intervening in preadolescent children with the intent to establish and maintain a healthy active lifestyle can be achieved, and there is evidence these effects can be maintained over time. The suspected mechanism of change is that the parents (or possibly day care providers) have complete control over the activity and eating environment in which children spend most of their time, and both daycare providers and parents serve as the most powerful role models for promoting healthy active living in their children. As such, there are evidenced based reasons to target day care providers and/or parents as intermediaries in intervention with young children in order to facilitate the adoption and maintenance of healthy active living behaviours by capitalizing on the dependent nature the children are at given their early stage of development. In fact, given preschoolers’ stage of development that results in a high degree of responsiveness to changes to the day care or home environment, there is reason to believe that intervening with day care providers and/or parents may produce even stronger effects of changes in healthy active living behaviours in preschoolers compared to those observed in older children.

It is important to note that PA and sedentary behaviours begin at a young age and have been shown to track from early childhood into adolescence and adulthood [71], which provides additional justification to target intervention in the preschool years. Thus, by getting young children more physically active and less sedentary, this may alter their PA trajectory and increase the likelihood that they will be physically active throughout development and into adulthood, thereby reaping the concomitant health benefits of an active lifestyle.

Our research group has recently completed a systematic review of PA in infants, toddlers and preschoolers [69] in relation to health and development to provide evidenced based PA guidelines for Canadian preschool aged children. In preschoolers, there was low to high quality evidence on the relationship between increased or higher physical activity and improved measures of adiposity, motor skill development, psychosocial health and cardio-metabolic health indicators. This review, intended to inform the development of Canadian PA recommendations for preschoolers, highlights the paucity of research that exists among 3–5 year old children and thus PA recommendations will be based on a limited, albeit growing body of evidence. The review also highlighted the need for randomized controlled trials to better identify the methods and procedures required to train childcare providers to maximize the health benefits of PA within the daycare setting.

## 6. Methodological Description of Our Pilot RCT Aimed at PA Promotion in Preschoolers

To our knowledge, we are the first group in Canada to implement a randomized controlled trial testing the efficacy of a lifestyle intervention designed to increase physical activity, motor skill development and improve body composition and reduce sedentary behaviour in preschool aged children attending licensed day care centres. Our current 2-armed randomized, controlled pilot trial (intervention daycare *vs.* control daycare) is designed to compare the effects of working with the day care providers to evaluate if the day care setting is a feasible and effective environment for changing health behaviours in preschool children, considering this is the environment in which many preschoolers spend the majority of their waking hours. Preschoolers are being evaluated at baseline, 3 months and 6-months post workshop intervention training (explained in more detail below). Our design includes an interim assessment at 3-months because some behavioural interventions decay over time while others take time for the full therapeutic effects to emerge. We have successfully assessed 72 children (40 intervention, 32 controls) from six day care centres (three intervention, three controls) spread over two cohorts. We have recently completed 3-month assessment in our second and last cohort. Over 80% of parents approached have consented to having their children participate in our trial, and we have obtained good compliance to the behavioural protocol (explained in more detail below in Lessons Learned section).

Our primary outcome variable is time spent in moderate to vigorous physical activity (MVPA) using the Actical accelerometer to objectively measure PA and sedentary behaviour. To account for the possibility that not all children wear the Acticals every day, and the length of time that children will wear them differ from day to day, the proportion of PA and sedentary behaviour per hour of wear time will be computed, as done previously in a group of preschool children [20]. Data will be collected in 15-second epochs and Pfeiffer *et al.*’s [93] cut points for 3 to 5 year old children’s PA intensity will be applied to derive time spent at various intensities of movement. Research has shown that most young children in the age range we are targeting are amenable to wearing these monitors and produce valid measurements [62,93,94], thus we are expecting to eliminate very few children from the final analysis, consistent with our experience in our pilot study with preschoolers. Secondary objectives include how the intervention influences gross motor and fundamental movement skills and anthropometrics. Gross motor skills are being assessed by the widely used Test of Gross Motor Development-2 (TGMD-2) [95], which is a validated and standardized norm-referenced measure of 12 common gross motor skills of children ages 3 to 11 years [96]. In addition, height, body weight, body mass index (kg/m^2^) are being directly measured. Body composition (lean body mass, fat mass, percent body fat) is being assessed using a bioelectrical impedance analysis system that accommodates the small feet of young children and has been validated for preschool-aged children [97,98].

## 7. Overview of Behavioural Intervention Training Protocol to Day Care Providers

Our preschoolers activity trial pilot, designed to increase PA and reduce sedentary behaviour in 3–5 year old children, includes, in brief, training workshops for daycare-providers focusing on the importance of, and strategies for implementing a variety of structured and unstructured physical activities, and reducing sedentary behaviour. The workshops focus on the importance of PA and movement skills for pre-school-aged children, understanding structured and unstructured play, familiarization with the intervention tools and broad practicalities of implementing a physical activity program in daycare centres, and include demonstrations of activities related to movement skills. The training workshops also assist providers with overcoming barriers to facilitating PA; understanding the range of movement skills; and using everyday materials to facilitate PA and active play. In addition, each daycare centre in the intervention group is provided with a resource training manual, recommended activity program outline with log sheets, music developed for physical activity with an outlined guidebook and starter kit of equipment, which collectively forms the basis of training for the daycare providers and the intervention itself. The manual is compiled with various ways providers can get children active in structured and unstructured physical activities, some of which target motor skill development. The early childhood educators are provided with weekly schedules that suggest a set of activities, drawn from the manual, that can be incorporated each day with the aim of meeting the NASPE guidelines of not having children sedentary more than 60 minutes at a time, and that children accumulate at least 60 minutes in structured PA and at least 60 minutes of unstructured (active play) each day, preferrably at moderate intensity or higher. These suggested activities have been broken down as locomotor skills, manipulative motor skills, and moving to music, shapes and creative play. We have also implemented follow-up support or ‘booster’ sessions which take place during regular hours within intervention day care centres. These multi-dimensional, in-centre, project staff-guided, structured PA sessions engage both the preschoolers and providers, and include performance monitoring and feedback related to implementation successes and, where relevant, include overcoming barriers, troubleshooting and problem solving.

## 8. Lessons Learned from Implementing a Behavioural Intervention in Day Care Setting

### 8.1. Practical/Applied Issues

Based on our experience, this type of intervention presents some notable challenges. First and foremost, day care provider engagement and personal investment in the trial can vary greatly. Despite the fact that the day care administration may be keen to participate in an intervention and trial, individual day care providers interacting with the participating children have personal attitudes and perceptions regarding how much they value PA which may impact the quality and quantity of the activities that they facilitate as part of the intervention. Day care provider enthusiasm for the administration of the intervention and willingness to comply with testing procedures may also affect efficiency and effectiveness in administering this type of trial. Their underlying knowledge may also be a factor as we have found that some of the providers with a greater pre-existing knowledge of general health considerations were more likely to adapt to the intervention protocol at the onset. The underlying purpose of this intervention is to provide evidence that intervening in preschool years can improve child health, thereby providing an evidence base to support a change in child care regulatory agency policies that influence preschool programming to ones that foster healthy active behaviour. To do so, the training and education (degree programs, certification *etc.*) of early childhood educators would need to be modified to ensure they have the required knowledge and skills for promoting and engaging young children in physical play, reducing sedentary time and facilitating motor skill development as has been suggested by the most recent Institute of Medicine—Early Childhood Obesity Prevention Policies report [99]. 

Seasonal issues can also arise whereby children may be away on vacation during testing conducted in the summer months and presumably this may also be a factor if testing is planned in December around the holiday period. To coincide with this, our experience in conducting our pilot study suggests the attrition rates can be inflated due to children changing daycares/moving, which often occurs in the summer months.

## 9. Lessons Learned for Research Using Behavioural Intervention in Day Care Settings

As a result of our experience conducting a RCT in daycare centres, we have several considerations and suggestions for conducting a trial of similar nature in the preschool population:

(1) If ethics boards will allow, have a direct line of contact with the parents provided on the consent form in an attempt to bypass communication to the parents through the day care provider. This may mitigate the confusion of messages being passed through a third-party member.
(2) Ensure all assessment equipment is “child friendly”. Time should be made for equipment adaptation (*i.e.*, placing stickers upon electrodes to make the Bioelectrical Impedance Analyzer system used to assess body composition less intimidating).
(3) The easier the better. Ensure the physical activity program is as straight forward as possible to enable compliance.
(4) If offering supplemental “booster” sessions in an effort to provide educational reinforcement on a regular basis, we would advise that they are standardized across the duration of the intervention timeline and we would also suggest that the more that can be offered, the better. Providing assistance on overcoming barriers to protocol implementation during these booster sessions may be a critical strategy for maximizing compliance and minimizing attrition.
(5) If conducting a similar trial at multiple centres during the same period, we would recommend that testing of control and intervention settings be performed on the same day to account for daily variations in weather, time of day, day of the week, *etc.*
(6) Outcome assessments should be organized as to not coincide with summer or winter holidays in order to maximize retention at each testing interval.
(7) Use consistent and frequent positive reinforcement with daycare providers as well as children to enhance morale and increase the likelihood of compliance.

## 10. Conclusions and Directions for Future Research

Child obesity and inactivity have reached epidemic proportions resulting in a public health crisis in our youngest citizens. In contrast to widely held societal and parental perception, initial epidemiological research indicates the high rate of obesity and sedentary behaviour are almost as prevalent in preschool aged children as they are in older children and adolescents.

The health benefits of increased PA are well documented. Moreover lifestyle behaviours and obesity are more difficult to modify over the long term in older youth [100] and adults [101]. Given physical activity and inactivity habits track from early childhood through development into adulthood [71], promoting PA in the preschool years may represent a critical period to intervene to optimize health promotion and chronic disease prevention. To support this recommendation, future research utilizing prospective randomized controlled trials with multiple follow-up assessments aimed at establishing and maintaining a healthy active lifestyle in preschoolers is of paramount importance. In addition, given parents are arguably the most powerful role models and control young children’s eating and activity environment, these trials should evaluate the incremental benefits of including parents and the home environment in these intervention studies, an area of inquiry that remains uninvestigated. Future research should also examine adherence to behavioural protocols, providers’ attitudes, perceptions and beliefs and intentions to implement PA in the daycare, and other aspects that would inform potential processes of behaviour change in preschool aged children.

Given young children spend the majority of their waking hours in child care, intervening with providers in the daycare setting may be an ideal environment for the promotion of PA and motor skills and its associated health benefits. Public health policies and legislation regarding day care environments differ widely within and between countries and cities. From a public health perspective, training of daycare providers and early childhood educators should include a focus on the importance of PA promotion and its role in healthy child growth and development and chronic disease prevention. Furthermore, policies that promote positive change in PA and motor skills should be developed and implemented and their impact on the health of preschool aged children should be evaluated more widely and thoroughly. 
